# Spinal rosette-forming glioneuronal tumor: A case report

**DOI:** 10.1097/MD.0000000000018271

**Published:** 2019-12-10

**Authors:** Shuji Hamauchi, Mishie Tanino, Kazutoshi Hida, Toru Sasamori, Shunsuke Yano, Shinya Tanaka

**Affiliations:** a Department of Neurosurgery, Sapporo Azabu Neurosurgical Hospital, Hokkaido; b Department of Neurosurgery, Hokkaido University Graduate School of Medicine, Sapporo; c The Department of Surgical Pathology, Asahikawa Medical University, Asahikawa; d Department of Cancer Pathology, Hokkaido University Faculty of Medicine, Sapporo, Japan.

**Keywords:** intramedullary tumor, RGNT, spinal rosette-forming glioneuronal tumor, synaptophysin

## Abstract

**Rationale:**

Rosette-forming glioneuronal tumor (RGNT) is a rare tumor which has been first reported as the fourth ventricle tumor by Komori et al and is classified as a distinct clinicopathological entity by the WHO Classification of Tumors of the Central Nervous System as in 2007. Although RGNTs were reported to occur in both supratentorial and inflatentorial sites, only 4 case reports of spinal RGNT have been demonstrated.

**Patient concerns:**

A 37-year-old female presenting with slowly progressing right-sided clumsiness. Cervical magnetic resonance imaging revealed a spinal intramedullary tumor between the C2 and C5 levels.

**Diagnoses:**

Pathological analysis showed unique biphasic cellular architecture consisting of perivascular pseudorosettes dominantly with few neurocytic rosettes and diffuse astrocytoma component. The tumor cells composed of perivascular pseudorosettes showed positivity for both synaptophysin and glial markers such as GFAP and Olig2. Therefore, the diagnosis of RGNT was made.

**Interventions:**

Gross total resection of the tumor was achieved. No adjuvant chemotherapy nor radiotherapy was conducted after operation.

**Outcomes:**

At 2 years after the operation, no recurrence was observed.

**Lessons:**

Although RGNT arising from the spinal cord is extremely rare, we need to consider the tumor as a differential diagnosis for intramedullary spinal cord tumors.

## 1 Introduction

Rosette-forming glioneuronal tumor (RGNT) is a rare tumor usually arising from the forth ventricle. RGNT was first described in 1998 by Komori et al^[1]^ and later, they have reported as case series. It is now recognized as a new entity and was later classified as grade I tumor by World Health Organization. RGNT has 2 distinctive features: a glial component and a neurocytic component forming neurocytic rosettes and/or perivascular pseudorosettes. In addition, positivity of synaptophysin in the neurocytic component is important feature in the diagnosis of RGNT.^[2]^ Despite of these pathological characteristics, the diagnosis of RGNT is difficult mainly because of its rare incidence. RGNT most commonly arises from the forth ventricle but was reported to arise in the cerebellum, brain stem, pineal region, third ventricle, lateral ventricle, hypothalamus, and optic chiasm later.^[3]^ RGNT of the spinal cord origin is rare and to date, there have been only 4 case reports of spinal RGNT in the English literature.^[4–6]^ Although the molecular analysis of RGNT of the fourth ventricle revealed higher mutation in FGFR1 and PIK3AC, only 1 case of RGNT of spinal cord was previously analyzed about these mutations.^[5,7]^ We here showed the spinal RGNT case with radiological, pathological, and molecular findings with review of the literature. The patient has provided informed consent for publication of the case.

## 2 Case report

**Figure 1:**
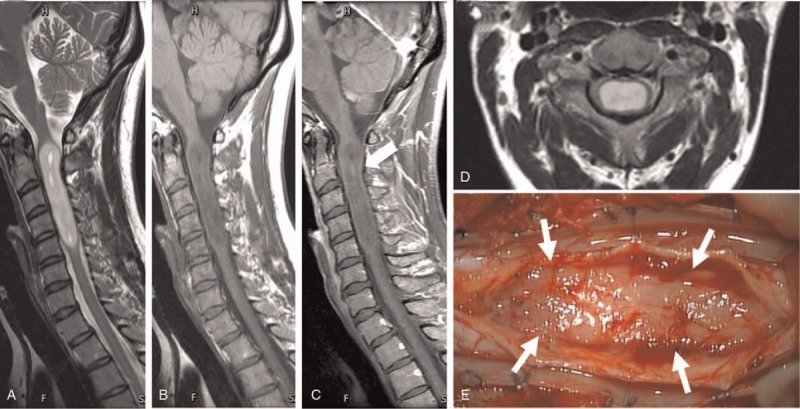
Magnetic resonance imaging (MRI) demonstrates the well-defined intramedullary tumor in the spinal cord from C1 to C5 level. (A and B) Sagittal images showing low intensity in T1-weight imaging, high intensity in T2 weight imaging. (C) Contrast-enhanced MRI showed a very slight enhancement at the C2 level (black arrow). (D) Axial image of T2-weighted MRI demonstrating lateralization of the tumor to right side. (E) Intraoperative picture following midline myelotomy is shown. Arrows indicate the tumor inside the spinal cord.

A 37-year-old female with no significant medical history and no significant family history presented with a 1-year history of slowly progressive right-hand clumsiness. Neurological examination revealed right-sided hemiparesis (manual muscle testing [MMT] score 4), hyperreflexia of the right upper and both lower extremities, and hyperalgesia below Th8 on the left side. Magnetic resonance imaging (MRI) showed the well-defined lesion in the cervical spinal cord (C2–5), which showed the low intensity with T1-weight imaging (Fig. 1A), high intensity with T2 weight imaging (Fig. 1B) and a very slight enhancement with gadolinium (Gd) at the C2 intradural level (Fig. 1C). The mass was slightly eccentric to the right side from the spinal cord center on the axial image (Fig. 1D). Several small cysts were also found in the tumor on the T2-weight imaging (Fig. 1B). There were neither hemosiderin cap nor syrinx in the adjacent spinal cord. Computed tomography showed no calcification in the mass (data not shown). The intraoperative gross appearance of the tumor with soft and gray color is shown in Figure 1E and the lesion was totally resected through a posterior midline myelotomy. The tumor had an adhesion to the tissue around the central canal at the C4 level although most of the boundary was easily peeled off.

**Figure 2:**
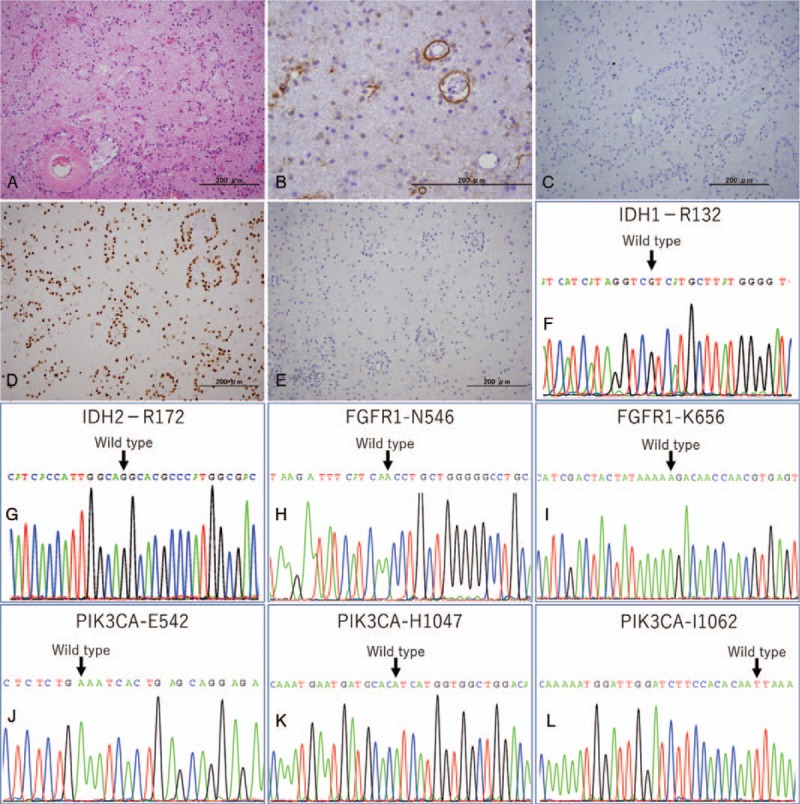
The histology of the present case. (A and B) Biphasic pattern consisting of neurocytic and glial component was observed. The neurocytic component consist of perivascular rosette formation had immunopositivity of synaptophysin. (C) The MIB-1 antibody stained 5% in the tumor nuclei. (D) Olig-2 is expressed in astrocytic component. (E) Neurocytic component negative for NeuN. (F–L) Sanger sequence tracing of IDH1/2, FGFR1, and PIC3CA. Black arrows indicate previously reported mutational hotspots of the genes in rosette forming glioneuronal tumor of the forth ventricle, all showing wild type sequences.

Pathologically, the tumor composed of cells with small, round nuclei showed perivascular pseudorosette where the tumor cells surrounded a small vessel with little nuerocytic rosette and diffuse astrocytoma, which was diagnosed as a WHO grade I RGNT featured by the biphasic histopathology consisting of glial and neurocytic components (Fig. 2A). Both of mitotic activity and necrosis were absent. In immunohistochemistry (IHC), both neural marker such as synaptophysin (Fig. 2B) and glial marker such as GFAP and Olig2 (Fig. 2C) were strongly positive for perivascular pseudorosettes, but negative for NeuN (Fig. 2D), neurofilament protein, and epithelial membrane antigen (EMA). The rosenthal fibers, eosinophilic granular bodied, and mirocalcification were not evident even in glial component. MIB-1 labeling index (Ki-67) showed 5% in the tumor (Fig. 2E). As its atypical location, the case was further sent to an expert for second opinion, who confirmed the initial diagnosis of RGNT. Molecular analysis for hot spot mutations of IDH1/2, BRAF, FGFR1, and PIK3AC was performed using formalin-fixed paraffin embedded specimens. The DNA sequencing revealed wild type of IDH1/2 (Fig. 2F and G), FGFR1 (Fig. 2H and I), and PIK3CA (Fig. 2J–L), *BRAF* genes and fluorescence in situ hybridization showed no 1p/19q-co-deletion nor KIAA1549-BRAF fusion.

Postoperatively, right hemiplegia was worsened to MMT grade 3. After 4 months of rehabilitation, the patient's neurological symptoms were alleviated, and independent walking was achieved, although clumsiness of right hand and hypalgesia below neck were persistent. No recurrence of the tumor has been observed at the 2-year follow up.

## 3 Discussion

We have reported a case of spinal RGNT occurred in 35-year-old female with distinct clinicopathological features including unusual morphology and genetic results. RGNT is rare tumor which affects commonly fourth ventricles of young to middle-aged adults with female predominance and showed 2 distinctive morphologies: a glial component, whose morphology resembles pilocytic astrocytoma, and a neurocytic component forming neurocytic rosettes and/or perivascular pseudorosette.^[2,8]^ As the present case was characterized by predominant perivascular pseudorosette formations with few neurocytic rosettes and glial component, we considered RGNT showed wide range of glial to neurocytic/perivascular pseudorosette component. Pathologically, the initial histological examination by H&E staining impressed ependymoma; however, the immunohistochemistry (IHC) is much different from ependymoma. Synaptophysin and glial markers such as GFAP and Olig2 were positive in the perivascular pseudorosettes but negative for EMA. Neurocytic components in RGNT have reported synaptophysin positivity in IHC but negative for GFAP. However, Chakraborti et al demonstrated both synaptophysin and GFAP in the neurocytic component of RGNT in immunohistochemistry and confocal analysis showed the positivity for stem cell marker, CD133. They suggested the RGNT originated from the progenitor cells of the subependymal plate which could differentiate biphenotypically.^[9]^ Duan et al reported 2 cases of spinal RGNT and documented that the tumors were found to originate from central canal of spinal cord.^[6]^ As our case also suggested the central canal origin tumor during operation, we suspected this tumor might originate from the progenitor cells of the subependymal cells in the central canal of spine, which can differentiate both glia and neuron.

RGNT commonly arises from the forth ventricle; however, 4 case reports of spinal RGNT have been described in the English literature,^[5–6,8]^ and the current case is the fifth case report (Table 1). The age is between 26 and 44 years (average 35 years) and there is a female predominance (male:female = 1:4) similarly to the past reports of RGNT of the fourth ventricle. Almost all cases except 1 case occurred in cervical spinal cord and the other case involved in thoracic to lumber spinal cord. MRI revealed cystic and solid intramedullary lesion with hypoisointense on T1-weighted image, hyperintense on T2-weighted image, and various enhancement patterns from heterogeneous to ring enhancement on Gd contrast. These features on MRI were consisted with RGNT of intracranial origin. Syringomyelia in the spinal cord adjacent to the tumor has been also reported in the literature similar to ependymoma and astrocytoma arising from the spinal cord. Hemosiderin deposition in the spinal cord adjacent to the tumor, which is not rarely observed in ependymoma, has been reported in 1 case. Unlike intracranial RGNT, satellite lesions on MRI and calcifications on CT scans have not been reported in spinal RGNT. Our case seemed to show weaker enhancement than documented in previous reports and neither syringomyelia nor hemosiderin deposition was observed on MRI.

**Table 1:**
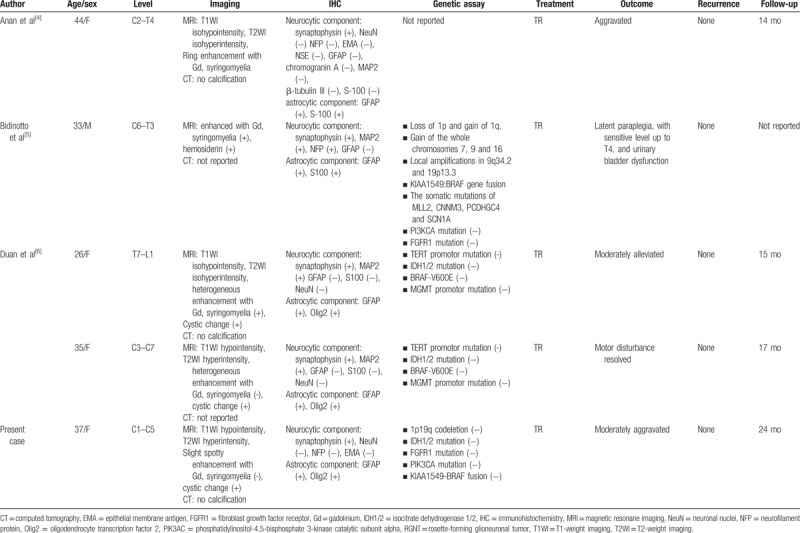
Summary of reported cases of RGNT arising from spinal cord.

The preoperative differential diagnosis of RGNT in the spinal cord includes ependymoma, low-grade astrocytoma, and pilocytic astrocytoma.^[9]^ In our case, as the intensity of Gd enhancement was very slight, ependymoma was not likely and possibility of low-grade astrocytoma was also unlikely because of well-defined boundaries. We thought that PA could not be denied because of its clear margin with weak enhancement on Gd contrast MRI; however, this patient's age was relatively older than the predilection age.^[10]^ As the spinal RGNT is very rare and shows relatively similar neuroimaging characteristics with other intramedullary tumors such as ependymoma and PA, the preoperative diagnosis is difficult.

There are still limited number of reports about the genetic characteristics of RGNT. Gessi et al first reported FGFR1 mutations in RGNT of the fourth ventricle, hypothesizing the molecular similarities between RGNT and PA.^[11]^ Kitamura et al have reported FGFR1 and PIK3CA mutations were found 46.2%and 44.4%, respectively and these mutation were detected in both glial and neurocytic components of RGNT.^[7]^ Among the 4 spinal RGNT cases, Bidinotto et al only examined hotspot mutations of FGFR1and PI3KCA; however the hotspot mutations of them were not detected.^[5]^ We also failed hot mutations of FGFR1, PIK3AC, BRAF, and IDH1/2 hotspot mutations. Although we do not have enough data of the molecular results of spinal RGNT, the genetic features of spinal RGNT might be different from the fourth ventricle origin.

Surgical resection is reported to be the first choice for the treatment of spinal RGNT and no adjuvant therapy can be recommended. In intracranial RGNT, recurrence-free survival rates have been reported to be 92.9% at a mean follow-up period of 22.5 months.^[12]^ All spinal RGNTs underwent complete resection without recurrence. As the rarity of spinal RGNT, it may be misdiagnosed as ependymoma or astrocytoma without immunohistochemistry; however, both clinical information including the patient's age, sex, and the tumor location and pathological findings such as biphasic pattern consists of neurocytic and glial components lead to narrow the differential diagnosis. It is important to accumulate spinal RGNT cases to reveal the true nature including molecular features and clinical course of the disease.

## Author contributions

**Conceptualization:** Mishie Tanino, Kazutoshi Hida.

**Data curation:** Shuji Hamauchi, Toru Sasamori, Shunsuke Yano.

**Methodology:** Mishie Tanino.

**Supervision:** Shinya Tanaka.

**Writing – original draft:** Shuji Hamauchi.

Shuji Hamauchi orcid: 0000-0003-2760-5897.
